# Laparoscopic median arcuate ligament release using an anterior approach for median arcuate ligament syndrome

**DOI:** 10.1002/ags3.12858

**Published:** 2024-09-10

**Authors:** Koji Kubota, Akira Shimizu, Tsuyoshi Notake, Satoshi Nakamura, Yuji Soejima

**Affiliations:** ^1^ Division of Gastroenterological, Hepato‐Biliary‐Pancreatic, Transplantation and Pediatric Surgery, Department of Surgery Shinshu University School of Medicine Nagano Japan

**Keywords:** celiac artery, inferior phrenic artery, laparoscopic median arcuate ligament release, median arcuate ligament syndrome, stenosis

## Abstract

Median arcuate ligament syndrome (MALS) is a rare condition characterized by nonspecific symptoms, such as abdominal pain, nausea, and vomiting. Furthermore, the development and rupture of pancreaticoduodenal artery aneurysms pose a potentially fatal risk. Median arcuate ligament release (MALR) is useful in the treatment of MALS, with most procedures performed laparoscopically. However, detailed descriptions of laparoscopic MALR (lap‐MALR) procedures are rare. In this study, we performed lap‐MALR via an anterior approach with dissection of the right lateral wall of the celiac artery (CA). For optimal visualization of the right side of the CA, the right branch of the inferior phrenic artery was divided. We believe that this procedure allows the MAL to be released within a sufficient surgical field and without excess or deficiency. Here, we present the details of six patients who underwent lap‐MALR for varying indications; three for pancreaticoduodenal artery aneurysms due to CA obstruction (unruptured, *n* = 1; ruptured, *n* = 2), two cases prior to hepato‐biliary‐pancreatic surgery, and one symptomatic case. In all cases, lap‐MALR was performed as described above, and the CA stenosis was successfully released. Our case series demonstrates the safety and reliability of our lap‐MALR procedure in the treatment of MALS‐related disorders, including pancreaticoduodenal artery aneurysms associated with CA compression.

## Graphical abstract

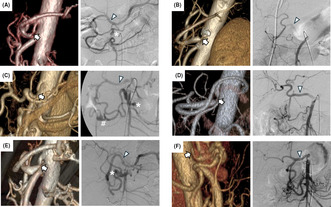

## 1 INTRODUCTION

Median arcuate ligament syndrome (MALS) refers to stenosis or occlusion of the celiac artery (CA) caused by the MAL and organ ischemia due to reduced blood flow in the CA region.^1^, ^2^ In some asymptomatic cases, collateral blood flow from the superior mesenteric artery via the pancreaticoduodenal artery (PDA) compensates, and blood flow in the CA region is maintained. However, increased PDA blood flow might lead to the development of a pancreaticoduodenal artery aneurysm (PDAA). PDAA rupture is sometimes fatal.^3^ Laparoscopic MAL release (lap‐MALR) is effective in MALS,^2^, ^4^, ^5^ and many cases have been reported^6^, ^7^, ^8^; however, reports detailing the surgical techniques are rare.^9^ The identification of the CA origin is often difficult owing to its anatomy. Furthermore, inadequate ligament dissection due to inappropriate surgical techniques in a narrow surgical field may not adequately improve blood flow to the CA region. There are typically two approaches to reach the CA origin: the anterior approach through the abdominal cavity and the retroperitoneal approach via the retroperitoneum,^8^ with each route having advantages and limitations. In this report, we outline the specific details of lap‐MALR performed using the anterior approach at our institution.

## 2 PATIENTS AND SURGICAL TECHNIQUE

### 2.1 Patients

**FIGURE 1:**
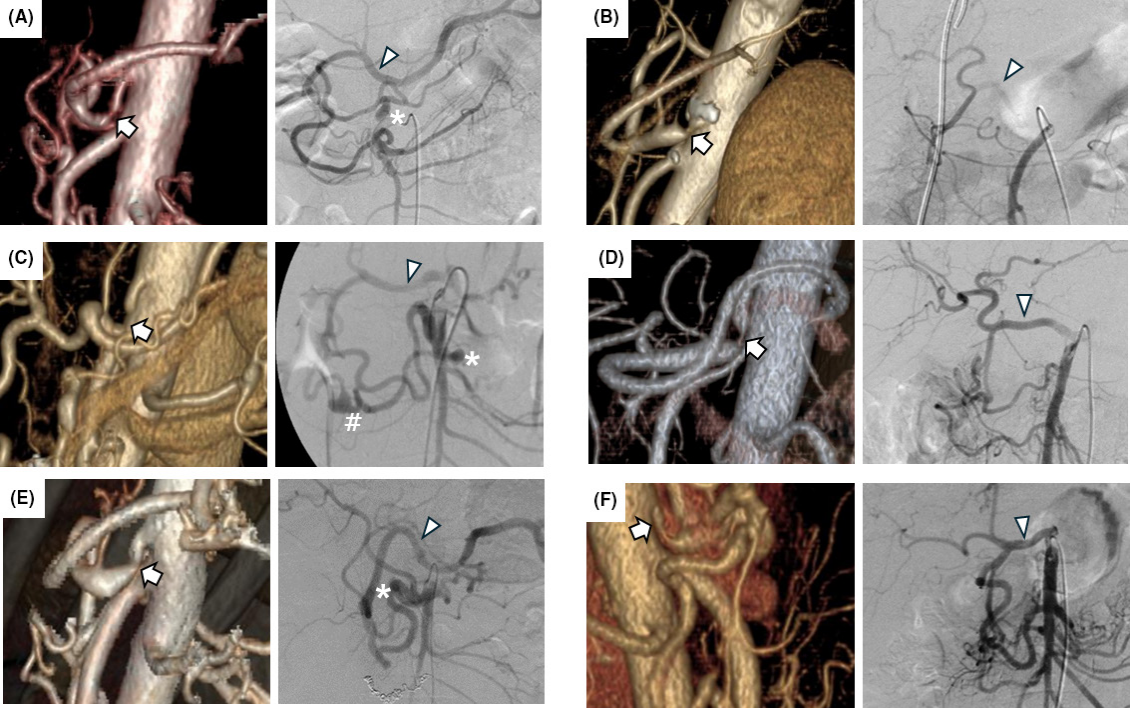
Characteristics of median arcuate ligament syndrome. Left panels, 3D CT‐angiography showing the celiac artery stenosis or obstruction (arrow). Right panels, angiography from the superior mesenteric artery. Retrograde contrast material was observed in the common hepatic arteries in all cases (arrowheads). (A) Case 1: Reflux to the level of the splenic artery. A pancreaticoduodenal artery aneurysm is visible (asterisk). (B) Case 2: Stents have been inserted for biliary obstruction owing to carcinoma of the papilla of Vater. (C) Case 3: A ruptured aneurysm is visible (indicated by #). (D) Case 4. (E) Case 5: After coiling for hemostasis. Reflux to the level of the splenic artery is visible, with a pancreaticoduodenal artery aneurysm (asterisk); (F) Case 6: Donor patient's findings prior to living donor liver transplantation. 3D, three‐dimensional; CT, computed tomography.

Six patients who underwent lap‐MALR via an anterior approach at our institution between November 2022 and October 2023 were included. The clinicopathological and demographic characteristics of these patients are summarized in Figure 1 and Table 1, respectively.

**TABLE 1:** Patient characteristics and surgical and short‐term outcomes.

Parameter	Total (*n* = 6)
Background characteristics	
Age, years^a^	54 (36–75)
Sex	
Male	3 (50)
Female	3 (50)
BMI, kg/m^2^ ^a^	22.1 (19.2–24.9)
Symptoms	
None	3 (50)
Rapture of PDAA	2 (33)
Abdominal pain	1 (17)
Preoperative comorbidity	
Hypertension	1 (17)
Diabetes mellitus	0 (0)
Respiratory disease	0 (0)
Cerebrovascular disease	0 (0)
Social history	
Smoking	1 (17)
Alcohol consumption	0 (0)
PDAA, yes	3 (50)
Rupture	2 (33)
Distance from the aorta to the stenosis, mm^a^	9.75 (3–12.5)
Stenosis length, mm^a^	4.6 (4–6)
Stenosis rate, %^a^	80 (57–100)
Pancreas – stenosis – aortic angle, degrees^a^	74.02 (38.6–153.07)
CA origin calcification, yes	1 (17)
Surgical outcomes	
Surgical time, min^a^	205.5 (162–284)
Blood loss, mL^a^	5 (5–80)
Postoperative hospital stay, day^a^	5.5 (5–8)
Improved blood flow, yes	6 (100)
Date of confirmation, POD^a^	3 (2–7)
Postoperative short‐term outcomes	
Major complication (≥CD grade IIIa)	0
Complication: CD grade I (post‐dural puncture headache)	1 (17)

*Note*: Values in parentheses are percentages unless indicated otherwise.

Abbreviations: BMI, body mass index; CA, celiac artery; CD, Clavien–Dindo classification; PDAA, pancreaticoduodenal artery aneurysm; POD, postoperative day.

^a^ Median (range).

### 2.2 Surgical technique

The procedure was performed under general anesthesia, with all patients placed in a supine split‐legged, reverse Trendelenburg position with approximately 10° of elevation. The operator stood to the right of the patient, and the camera operator stood between the patient's legs.

**FIGURE 2:**
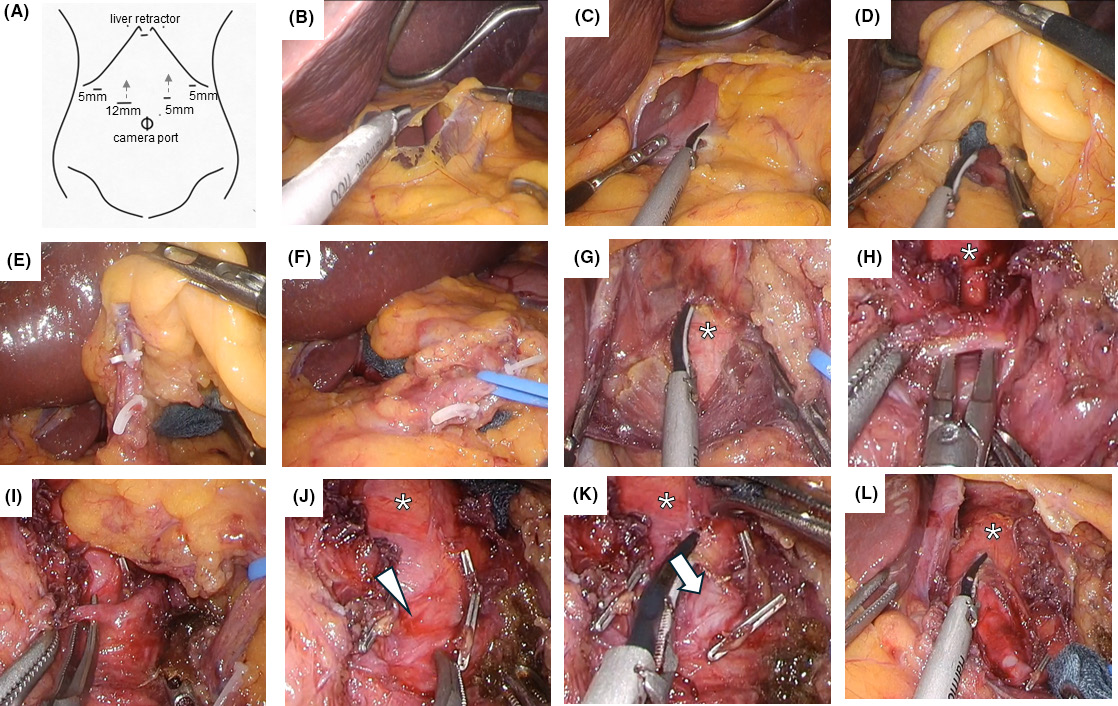
Details of the surgical procedure for laparoscopic median arcuate ligament release. (A) Port placement; (B) opening the lesser omentum; (C) the peritoneum of the muscle bundle of the right diaphragmatic crus is incised; (D) the pedicle containing the left gastric artery is mobilized from the crura; (E) the left gastric vein is dissected; (F) the left gastric artery is secured, and traction is applied; (G) the right and left crura are dissected to expose the surface of the aorta; (H) secured MAL with visible shiny, white bands; (I) secured right inferior phrenic artery; (J) once this artery is divided, the layer of tissue covering the CA becomes clearly visible (arrowhead); (K) the CA adventitia is exposed (arrow) to the level of the CA origin; (L) the aortic adventitia is exposed 3 cm from the origin of the CA. (G, H, J, K) The asterisk in each panel indicates the aorta. CA, celiac artery; MAL, median arcuate ligament.

- Port placement was based on the principles of laparoscopic distal gastrectomy. During manipulation of the CA origin, the left and right medial ports were moved towards the CA origin to ensure an optimal forceps angle. This facilitated subsequent manipulation. A 12‐mm port on the right medial side facilitated the attachment and removal of instruments and the insertion of a laparoscopic ultrasound probe for blood flow assessment (Figure 2A).
- A Nathanson hook liver retractor was inserted through the pericardial fossa, and the left lobe of the liver was elevated cephalad to ensure a clear surgical field. The hepatic branch of the anterior vagal trunk was preserved, and the lesser omentum was opened (Figure 2B).
- The peritoneum of the muscle bundle of the right diaphragmatic crus was incised (Figure 2C). The pedicle containing the left gastric artery was mobilized from the diaphragmatic crus (Figure 2D).
- The left gastric vein was dissected after clipping to prevent bleeding due to excessive traction during subsequent operations (Figure 2E). The left gastric artery was secured, and traction was applied, to expose the plexus to the anterior CA (Figure 2F).
- Dissection and division of the left and right diaphragmatic crura revealed the aortic surface near the CA origin (Figure 2G).
- Continued caudal traction on the left gastric artery exposed the MAL covering the CA. The MAL is a multilayered band containing muscles, ligaments, nerves, and blood vessels, which may appear shiny and white (Figure 2H). It is important to note that resection of this ligament (or, more accurately, part of the MAL) does not relieve compression of the CA.
- In cases where the inferior phrenic arteries (IPAs) branched from the CA, the right IPA was divided. Arterial bifurcation was assessed preoperatively. Once this artery was divided, the layer of tissue covering the CA was clearly visible (Figure 2I,J).
- This layer was carefully detached from the CA, exposing the CA adventitia to its origin (Figure 2K). The aortic adventitia was exposed 3 cm from the origin of the CA (Figure 2L).
- Intraoperative ultrasonography confirmed successful release if the direction of blood flow in the common hepatic artery changed and the velocity increased after MALR. However, intraoperative ultrasonography alone does not always provide a correct assessment of CA patency.
- An anti‐adhesion sheet was applied to prevent ligament reattachment, and no drains were inserted (Video S1).

**FIGURE 3:**
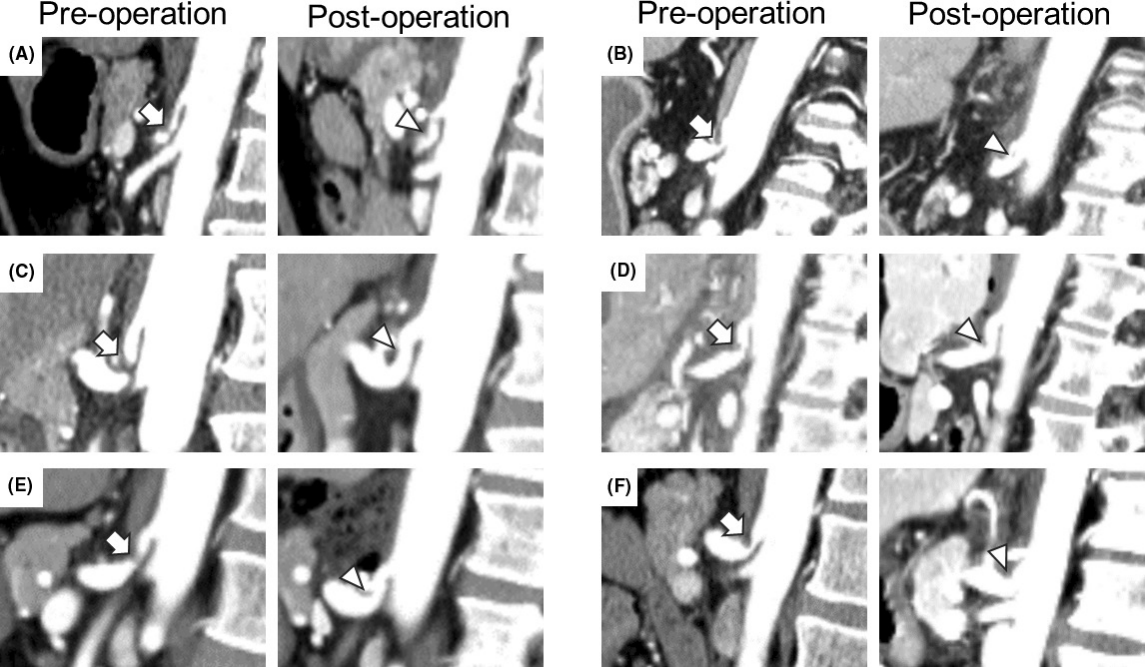
Postoperative assessment with contrast‐enhanced CT. Left panels, preoperative contrast‐enhanced CT showing stenosis or obstruction of the CA (arrow); Right panels, postoperative contrast‐enhanced CT showing dilation of the CA (arrowhead). (A) Case 1; (B) Case 2; (C) Case 3; (D) Case 4; (E) Case 5; (F) Case 6. CA, celiac artery; CT, computed tomography.

Contrast‐enhanced computed tomography (CT) was performed 2–3 days postoperatively to confirm CA patency (Figure 3). Additional interventions, such as interventional radiology with CA stent placement or aorto‐splenic artery bypass, were considered if improved CA blood flow was not achieved.

## 3 RESULTS

**FIGURE 4:**
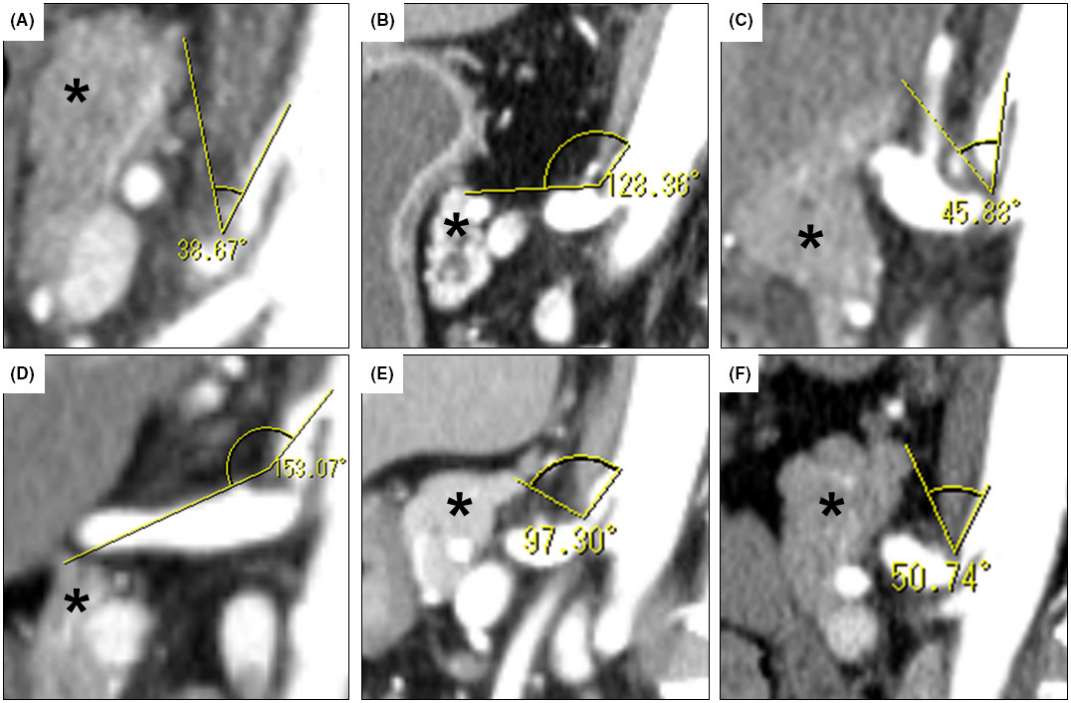
The angle between the cephalic edge of the pancreas and the CA origin, with the CA stenosis at the apex (asterisk). (A) Case 1; (B) Case 2; (C) Case 3; (D) Case 4; (E) Case 5; (F) Case 6. CA, celiac artery.

The patients' and MALS characteristics are shown in Figure 1 and Table 1, respectively. The median distance between the aorta and the CA stenosis was 9.75 mm (range, 3–12.5 mm), the median length of the CA stenosis was 4.6 mm (range, 4–6 mm), and the median stenosis rate (CA diameter − stenosis diameter/CA diameter) was 80% (range, 57%–100%). Calcification of the CA origin was observed only in Case 2, and PDAAs were observed in three cases, one of which was unruptured (Case 1) and two ruptured preoperatively (Cases 3 and 5) (Figure S1). In Case 1, the first stage of treatment was interventional radiology, followed by MALR. The median surgical time was 205.5 min (range, 162–284 min), and the median blood loss was 5 mL (range, 5–80 mL). No cases were converted to laparotomy, and no transfusions were performed. The maneuverability of forceps and overall surgical difficulty were notably influenced by the position of the cephalic edge of the pancreas relative to the CA stenosis. Preoperative prediction of surgical complexity was aided by measuring the angle formed by a line connecting the cephalic edge of the pancreas, the CA stenosis, and the CA origin using sagittal CT images (Figure 4). Blunt angles were easier to manipulate, whereas acute angles posed greater challenges. The median angle of stenosis was 74.02° (range, 38.67°–153.07°). Cases 1, 3, and 6 presented with acute angles, while Cases 2 and 4 featured blunt angles. The angle in Case 5 was nearly a right angle. Evaluation of CA blood flow was performed using contrast‐enhanced CT on postoperative day 2 or 3. Blood flow improvement was observed in all cases (Figure 3). The only postoperative complication was post‐dural puncture headache (Clavien–Dindo grade I) in Case 2. The median postoperative hospital stay was 5.5 days (range, 5–8 days). The median follow‐up was 9.5 months (range, 4–15 months), with no MALS recurrence in any patient.

## 4 DISCUSSION

MALR is a promising treatment option for patients with MALS, but owing to the rarity of this disease, a standardized surgical technique has not been established. In this report, we provided detailed insights into the technique and outcomes of lap‐MALR in six cases performed at our institution. Lipshutz^9^ determined from cadaveric anatomy that the origin of the CA overlapped the crura of the diaphragm. Harjola^10^ first performed MALR in 1963 and reported its efficacy. In 2000, the first lap‐MALR technique was reported by Roayaie et al.,^11^ and a subsequent meta‐analysis demonstrated its efficacy.^2^, ^4^ Nevertheless, the surgical details involved in performing this technique have not yet been established. Reported lap‐MALR approaches include an anterior approach from the abdominal cavity^7^, ^12^, ^13^ and a retroperitoneal approach.^8^ However, most studies have reported using anterior approaches; therefore, currently, this appears to be the main approach. With an anterior approach, it is important to consider the angle between the pancreatic cephalic edge and the CA origin, with the CA stenosis as the apex, because the position of the pancreas makes it difficult to observe and work with the CA stenosis. In Cases 2, 4, and 5, where the angle was obtuse, manipulation of the CA stenosis was relatively easy, whereas in Cases 1, 3, and 6, where the angle was acute, this manipulation was difficult (Table S1, Video S2). In cases in which manipulation is deemed difficult preoperatively, a retroperitoneal approach may be considered. More case studies involving the retroperitoneal approach are needed.

Preoperative identification of the bifurcation pattern of the IPA is very important to prevent intraoperative arterial injury, and the bifurcation is a good anatomical benchmark for the start of the dissection. Whitley et al. performed a detailed analysis of the bifurcation pattern of the IPA and found various patterns.^14^ Preoperative diagnosis of the IPA pattern was achieved in all of our cases. The anterior approach can be performed from the central^7^ or left side^12^, ^13^ or, as in our technique, from the right side. The compression of the origin of the CA consists of several overlapping bands of muscle, ligament, and neurovascular components that must be repeatedly dissected. The advantages of the right‐sided approach are^1^ the right inferior phrenic artery can be easily processed and^2^ after dividing this artery, the operative field is clear, and the layer to be dissected can be easily identified. However, it is unclear which route is safest and easiest to complete, as few reports have described the technique in detail. Therefore, further studies are warranted. A summary of the three approaches is presented in Table S2.

Recently, various surgical procedures have been performed with robotic assistance and have been reported useful in MALR.^15^, ^16^, ^17^ However, in a cohort study by DeCarlo et al.,^18^ robot‐assisted MALR was associated with poorer outcomes than either open‐ or lap‐MALR. This is probably owing to the limited proficiency of surgeons with this evolving method. Widespread use of robot‐assisted surgery may result in major improvements in outcomes. However, at this stage, MALR is best reliably performed laparoscopically.

A limitation of this retrospective observational study is the limited number of cases from a single center, which may have affected our findings in terms of treatment efficacy and complication rates. The development of collateral vessels and rupture of the PDAA were observed in most cases; however, only one patient presented with symptoms, such as abdominal pain. Lap‐MALR is highly effective in improving blood flow; however, this technique cannot be evaluated in terms of symptomatic improvement because removal of the ganglion may be required to improve abdominal symptoms.^2^, ^19^ While lap‐MALR is performed infrequently, we consider that our case series shows this technique to be safe and reliable.

## AUTHOR CONTRIBUTIONS

Koji Kubota wrote and edited the manuscript. Akira Shimizu and Yuji Soejima conceived the study and performed a critical review. All authors participated in patient treatment and treatment planning, and approved this manuscript.

## FUNDING INFORMATION

This study did not receive any specific grants from funding agencies in the public, commercial, or non‐profit sectors.

## CONFLICT OF INTEREST STATEMENT

The authors declare no conflicts of interest for this article.

## ETHICS STATEMENT

Approval of the research protocol: The protocol for this research project has been approved by a suitably constituted ethics committee of the institution and it conforms to the provisions of the Declaration of Helsinki. Ethics Committee of Shinshu University (6138).

Informed consent: Written informed consent was obtained from all study participants.

Registry and the registration No. of the study/trial: N/A.

Animal studies: N/A.

## Supporting information


Figure S1.

Table S1.

Table S2.

Video S1.

Video S2.

## Data Availability

Data supporting the results of this study are available upon request from the corresponding author.
