# Supplementary figures and images for: Laparoscopic median arcuate ligament release using an anterior approach for median arcuate ligament syndrome
Source: Ann Gastroenterol Surg. 2024 Sep 10;8(6):1137–43. doi: 10.1002/ags3.12858 (PMC11533021; doi:10.1002/ags3.12858)

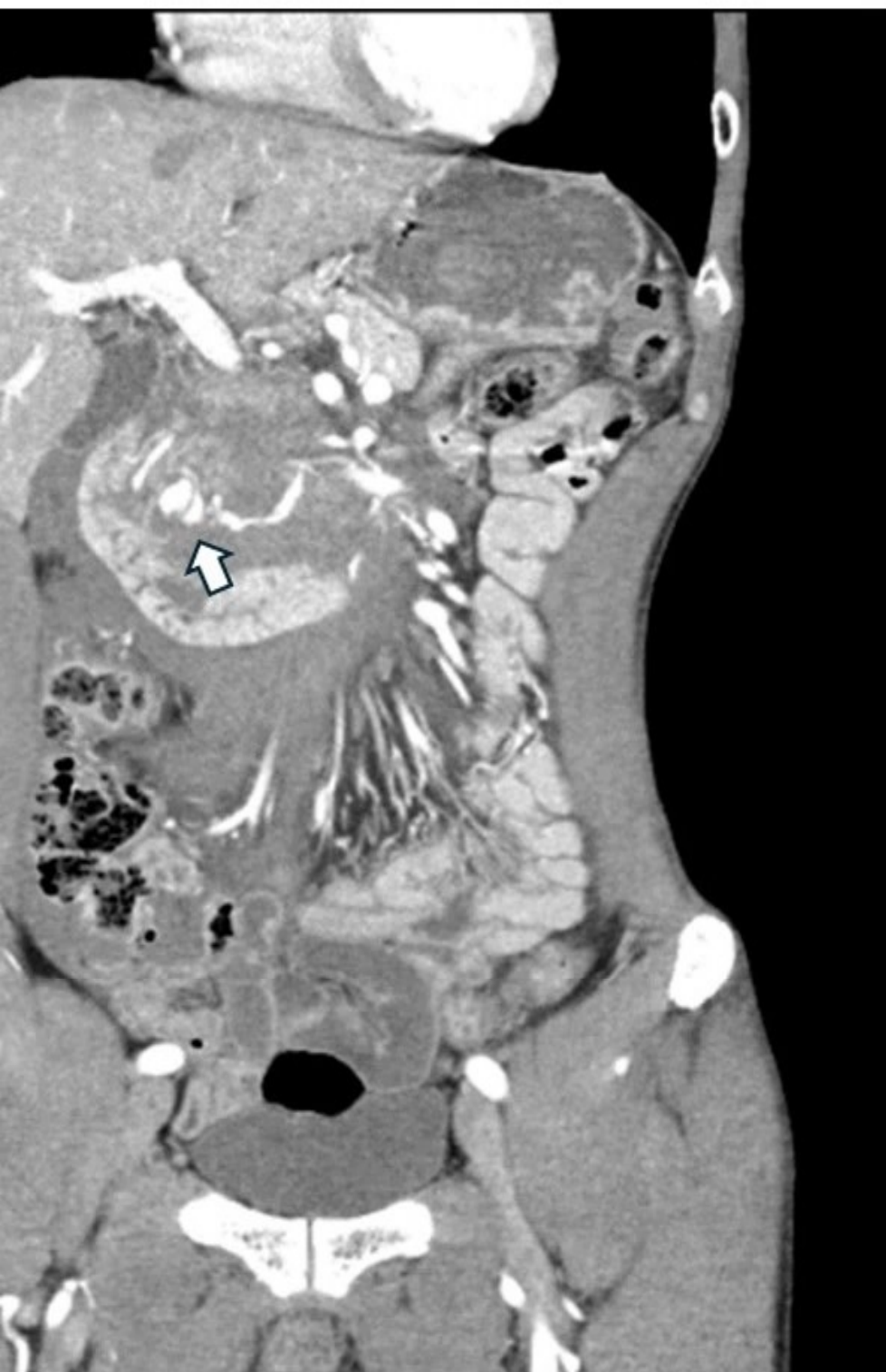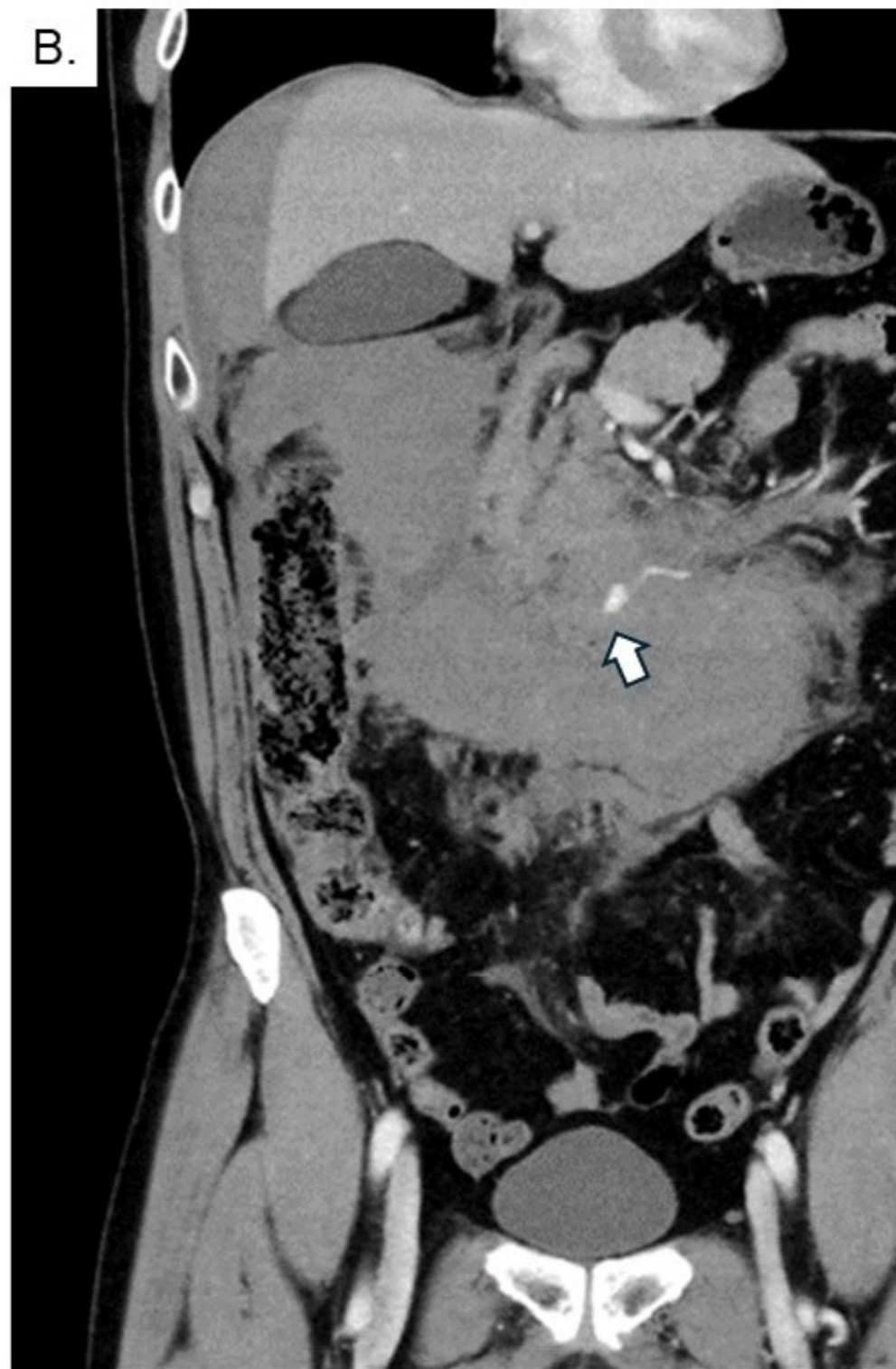

Supplement: Supplementary file 1 — Figure S1. [file AGS3-8-1137-s001.pdf]
