# Supplementary material for: Laparoscopic median arcuate ligament release using an anterior approach for median arcuate ligament syndrome
Source: Ann Gastroenterol Surg. 2024 Sep 10;8(6):1137–43. doi: 10.1002/ags3.12858 (PMC11533021; doi:10.1002/ags3.12858)
Supplement: Supplementary file 2 — Table S1. [file AGS3-8-1137-s003.docx]

**Supplementary Table 1.** Comparison of the patients’ characteristics in the acute angle group and obtuse angle group

| Characteristic | Acute angle group n=3 | Obtuse angle group n=3 | p-value |
| --- | --- | --- | --- |
| Age, median (range) | 48 (36–51) | 60 (57–75) | 0.004 |
| Sex, Male/Female, n (%) | 2 (67)/1 (33) | 1 (33)/2 (67) | 0.410 |
| BMI, kg/m^2^, median (range) | 24.91 (19.16–27.33) | 21.22 (19.76–23.01) | 0.278 |
| Angle between the cephalic edge of the pancreas and the CA origin, °, median (range) | 53.5 (37.5–55.7) | 101.6 (87.4–125.2) | 0.004 |
| PDAA, yes, n (%) | 1 (33) | 1 (33) | 0.410 |
| PDAA rupture, yes, n (%) | 1 (33) | 1 (33) | 1.000 |
| Distance from the Ao to the stenosis, mm, median (range) | 10 (5–12.5) | 9.5 (3–17) | 0.860 |
| Stenosis length, mm, median (range) | 4.5 (4.4–6) | 4.7 (4–5) | 0.424 |
| Stenosis rate, %, median (range) | 100 (82.2–100) | 77.4 (57.3–77.8) | 0.004 |
| Operation time, min, median (range) | 211 (192–284) | 200 (162–228) | 0.268 |
| Blood loss, mL, median (range) | 15 (5–80) | 3 (3–5) | 0.019 |
| Transfusion, n (%) | - | - | 1.000 |
| Hospital stay, days, median (range) | 5 (4–7) | 6 (5–8) | 0.351 |

BMI, body mass index; CA, celiac artery; PDAA, pancreaticoduodenal artery aneurysm; Ao, aorta
