# Supplementary material for: Laparoscopic median arcuate ligament release using an anterior approach for median arcuate ligament syndrome
Source: Ann Gastroenterol Surg. 2024 Sep 10;8(6):1137–43. doi: 10.1002/ags3.12858 (PMC11533021; doi:10.1002/ags3.12858)
Supplement: Supplementary file 3 — Table S2. [file AGS3-8-1137-s005.docx]

**Supplementary Table 2.** Summary of right-sided, central, and left-sided approaches in MALR

|  | Right-sided approach | Central approach | Left-sided approach |
| --- | --- | --- | --- |
| Approach | Lesser curvature | Lesser curvature | Omental bursa |
| Ao exposure | Early | Late | Early |
| CHA | - | Expose | Expose |
| LGA | Secured and traction | Secured and traction | Secured and traction |
| IPA | Right IPA is divided | Unknown | Right and left IPAs are divided |
| Method of dividing the tissue covering the CA | Layer-by-layer | One bundle | Layer-by-layer |

MALR, median arcuate ligament release; Ao, aorta; CHA, common hepatic artery; LGA, left gastric artery; IPA, inferior phrenic artery; CA, celiac artery
